# Publisher Correction: Dysregulation of ghrelin in diabetes impairs the vascular reparative response to hindlimb ischemia in a mouse model; clinical relevance to peripheral artery disease

**DOI:** 10.1038/s41598-020-73064-6

**Published:** 2020-10-13

**Authors:** Joshua P. H. Neale, James T. Pearson, Kate N. Thomas, Hirotsugu Tsuchimochi, Hiroshi Hosoda, Masayasu Kojima, Takahiro Sato, Gregory T. Jones, Adam P. Denny, Lorna J. Daniels, Dhananjie Chandrasekera, Ping Liu, Andre M. van Rij, Rajesh Katare, Daryl O. Schwenke

**Affiliations:** 1 Department of Physiology, School of Biomedical Sciences and HeartOtago, University of Otago, 270 Great King Street, Dunedin, 9018 New Zealand; 2 Department of Cardiac Physiology, National Cerebral and Cardiovascular Center Research Institute, Suita, Osaka Japan; 3 Biomedicine Discovery Institute and Department of Physiology, Monash University, Clayton, Australia; 4 Department of Surgical Sciences, University of Otago, Dunedin, New Zealand; 5 Department of Regenerative Medicine, National Cerebral and Cardiovascular Center Research Institute, Suita, Osaka Japan; 6 Molecular Genetics, Institute of Life Science, Kurume University, Kurume, Japan; 7 Department of Anatomy, School of Biomedical Sciences, University of Otago, Dunedin, New Zealand

Correction to: *Scientific Reports* 10.1038/s41598-020-70391-6, published online 12 August 2020

In the original version of this Article, Rajesh Katare and Daryl O. Schwenke were omitted as equally contributing authors.

In addition, Rajesh Katare was omitted as a corresponding author. Correspondence and requests for materials should also be addressed to rajesh.katare@otago.ac.nz.

These errors have now been corrected in the HTML and PDF versions of the Article.

